# Chronic unpredictable mild stress induced depression-like behaviours and glutamate-glutamine cycling dysfunctions in both blood and brain of mice

**DOI:** 10.1080/13880209.2019.1598445

**Published:** 2019-04-16

**Authors:** Ya-Ping Chen, Chuang Wang, Jiang-Ping Xu

**Affiliations:** a School of Pharmaceutical Sciences, Southern Medical University, Guangzhou, PR China;; b College of Pharmacy, Fujian University of Traditional Chinese Medicine, Fuzhou, PR China;; c Ningbo Key Laboratory of Behavioral Neuroscience, Ningbo University School of Medicine, Ningbo, PR China

**Keywords:** CUMS, Glu, Gln, GABA, HPLC

## Abstract

**Context:** Currently, there is no cure or early preclinical diagnostic assay available for depression. Recently, depression has been observed in association with metabolic abnormalities of the glutamate (Glu)–glutamine (Gln) cycling, which is regulated by Glu, Gln and γ-aminobutyric acid (GABA) amino acids.

**Objective:** The purpose of this study is to determine the changes of Glu, Gln and GABA in blood and brain of chronic unpredictable mild stress (CUMS) induced mice and to clarify the depression biomarkers in the Glu–Gln cycling.

**Materials and methods:** Male Kunming mice were divided into model group and control group randomly (*n* = 12). The depression model of mice was established by CUMS stimulation for 56 days. The liquid chromatography-fluorescence method was used for simultaneous determination of Glu, Gln and GABA in the plasma and brain of mice. *o*-Phthalaldehyde and β-mercaptoethanol were used as pre-column derivatization reagents. Neurotransmitters were analysed on high performance liquid chromatography (HPLC) on an HPH C18 column in combination with a fluorescence detector.

**Results:** The method was simple, highly sensitive and showed excellent linearity with regression coefficients higher than 0.999, good accuracy (95–108%) and good inter-day precision (RSD <15%) for all analytes. Limit of quantification (LOQ) values were established as 0.01, 0.01 and 0.005 μg/mL for Glu, Gln and GABA. The GABA in the CUMS mouse brain (*p* < 0.01) was significantly increased and Gln in plasma (*p* < 0.01) and brain (*p* < 0.01) were both decreased.

**Conclusions:** Our study demonstrates that the Gln in plasma can be used as a biological marker of depression.

## Introduction

Major depressive disorder (MDD) is a serious mental disorder characterized by high illness rate, high crippling rate, high recrudescence rate and high suicide rate, affecting more than 300 millions of people worldwide. Depression is ranked by the World Health Organization (WHO) as the single largest contributor to global disability (7.5% of all years lived with disability in 2015) (WHO 2017). As the number of people suffering from MDD steadily increases, so too does the burden of accurate diagnosis. Currently, diagnosis of MDD requires clinical assessment by professionals with significant clinical experience. However, the inter-clinician variability of these assessments makes it difficult to track drug efficacy in clinical trials. A convenient and automated method to assess depression severity would both simplify and standardize the task of diagnosing and monitoring depression, allowing for greater availability and uniformity in assessing depression.

The monoamine hypothesis of depression has been the dominating pathophysiology of depression, and monoaminergic systems is also deemed as the main target of pharmacological treatments for depression in the last decades, but available antidepressants require 3–8 weeks to produce a therapeutic response though the monoamine neurotransmitters are immediately affected (Machado-Vieira et al. 2008). The critical pathogenesis of depression is still unclear. It is now widely accepted that drug discovery must move beyond the monoamine systems to improve patient outcomes. In particular, the glutamate (Glu) system has emerged as a vibrant area of depression research (Abdallah et al. 2015; Murrough et al. 2017).

Glu has an important role as the major excitatory neurotransmitter in the central nervous system. Glu is synthesized from glutamine (Gln) by glutaminase (GS) in neurons, then released into the synaptic cleft to exert biological effects. Released Glu is taken up by surrounding astrocytes *via* the Glu transporters, where it is metabolized to Gln by the mainly astrocyte-located GS, transported back to presynaptic neurons and reconverted to Glu. In addition, Glu also can be metabolized to γ-aminobutyric acid (GABA) by glutamic acid decarboxylase (GAD) (Hashimoto et al. 2016). Thus, the Gln–Glu–GABA cycle as one part of glia-neuron communication has an important role in excitatory and inhibitory neurotransmission. Many studies have shown that abnormality in Glu–Gln cycle plays an important role in the pathophysiology of depression (Chandley et al. 2014; Hashimoto et al. 2016).

However, the change of Glu levels in depression patient brain was not clarified. Hashimoto et al. (2007) reported that increased levels of Glu in the prefrontal cortex in postmortem brain samples from MDD and bipolar disorder, suggesting a role of the glutamatergic system in mood disorders. Other proton magnetic resonance spectroscopy (^1^H-MRS) studies found decreased levels of Glx (Glu and Gln) in the anterior cingulate cortex (Auer et al. 2000) and dorsomedial/dorsal anterolateral prefrontal cortex (Hasler et al. 2007) in patients with MDD. In addition, Godlewska et al. (2015) also found that there was no difference in Glu levels in the occipital cortex between MDD patients and controls. Thus, the results of studies on levels of Glu and Gln in depression patients are inconsistent. It is necessary to ascertain the exact changes of Glu in depression, and the other two amino acids in Glu-Gln cycling should also be detected to clarify the relationship with depression. So it is of great interest to examine levels of three key amino acids in Glu–Gln cycling which related with depression.

This study was designed to develop a high performance liquid chromatography (HPLC)-FLD method for simultaneous determination of the Glu–Gln cycling amino acids in both brain and plasma, and find a preclinical indicator in blood which can reflect the level in brain to diagnose depression.

## Materials and methods

### Reagents

Glu (98%, lot no. 140690-200401) was purchased from National Standard Material Centre (Beijing, China); Homoserine (IS) (99%, lot no. 5005F23A) was purchased from Alfa Aesar Chemical Co. Ltd (Shanghai, China); Gln (99%, lot no. BCBH4247V), GABA (97%, lot no. BCBN4574V), *o*-phthalaldehyde (OPA), β-mercaptoethanol, disodium hydrogen phosphate, sodium borate and perchloric acid were purchased from Sigma Aldrich Chemie (Steinheim, Germany). HPLC-grade acetonitrile and methanol were purchased from Merck (Darmstadt, Germany). Water was purified by means of a Milli-Q plus system from Millipore (Bedford, MA).

### Instrumentation

Chromatography was performed using the Agilent 1260 system equipped with a degasser (Agilent Technologies Santa Clara, CA) (model G1379), a quaternary pump (model G1311X), a temperature-controlled autosampler (model G1329B), column oven enabling temperature control of the analytical column (model G1316A) and a fluorescence detection (model G1321A). All chromatographic separations were performed on an Agilent Poroshell HPH-C18 column (4.6 mm × 50 mm, 2.7 μm).

### Derivatization procedure

The standards or samples were precolumn derivatized with OPA reagent solution. The derivatization reagent was: 27.0 mg OPA dissolved in 5 mL of methanol, and diluted with the 4.96 mL of borate buffer 0.4 M (pH 9.5) and 40 μL of β-mercaptoethanol, freshly prepared every week and protected from light exposure.

Derivatization of amino acids: A standard or sample was mixed with 5 μL of OPA derivatization reagent in the autosampler G1330B. The mixture was vortexed for 2 min before HPLC analysis.

### Animals

The adult male Kungming mice (6–8 weeks) used for the experiment were supplied by the Laboratory Animal centre of the Southern Medical University (Guangzhou, China) and Ningbo University (Ningbo, Zhejiang). The animals were housed in an air-conditioned room at 22 ± 3 °C and 60 ± 5% relative humidity under a 12 h light/dark cycle (lights on at 7:00 a.m.) with *ad libitum* access to food and water. All experiments were carried out in accordance with the principles of the ‘NIH Guide for the Care and Use of Laboratory Animals’ (NIH Publications No. 80–23, revised 1996). The procedures were approved by the Animal Care and Use Committee of the Southern Medical University.

### Chronic unpredictable mild stress (CUMS) procedure

This animal model of stress consisted of chronic exposure to variable unpredictable stressors, none of which was sufficient alone to induce long-lasting effects. Briefly, the CUMS procedure (Huang et al. 2017) involved 12 different stressors that were randomly arranged throughout the day and night over 56 consecutive days. The stressors were (1) 24 h of food deprivation, (2) 1 h of exposure to 4 °C room, (3) 24 h of exposure to a 45° cage tilt, (4) overnight illumination, (5) 24 h of exposure to a wet cage (100 mL of water per individual cage, which is enough to make the sawdust bedding wet), (6) 5 min of swimming in 6–8 °C water, (7) tail clamp for 5 min, (8) 24 h of water deprivation, (9) unpredictable shocks for 5 min (15 mA, one shock/5 s, 10 s duration), (10) swimming for 15 min, (11) 4 h of restricted movement and (12) 4 h of disrupting the cage. The behavioural tests were performed and scored by trained and experienced observers who were blinded to the animals’ conditions.

### Behavioural tests

**Figure 1.**
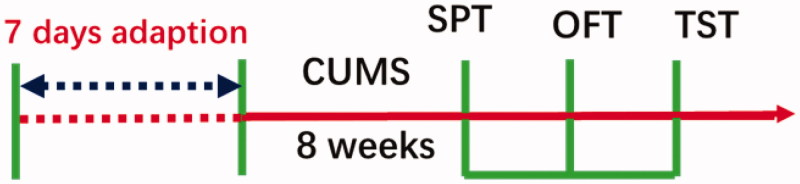
Experimental procedure.

The sucrose preference test (SPT) was conducted at the end of the CUMS procedure (week 8). The open field test (OFT) and tail suspended test (TST) were successively carried out after SPT. The behavioural tests were performed and scored by three trained and experienced observers. The experimental design of the study is depicted in Figure 1.

#### Sucrose preference test (SPT)

After eight weeks of CUMS stimulation, all animals were submitted to SPT. The SPT detected anhedonia-like behavioural changes and was with some modifications as previously described (Sharma and Thakur 2015). Before the test, mice were housed individually and habituated to a 1% sucrose for 24 h. Mice were deprived of water for 24 h then one bottle was filled with fresh water while another continued to contain 1% sucrose solution, followed by a preference test spanning 4 h. The positions of the two bottles were exchanged every 30 min during the test. After 4 h, the bottles were weighed and the consumed volume of sucrose and water was calculated. The sucrose preference was determined with the following formula: Sucrose preference (%) = sucrose consumption/(water consumption + sucrose consumption) × 100.

#### Open field test (OFT)

Briefly, the OFT was conducted according to the protocols reported recently (Li et al. 2017). The 50 × 50 cm arena with 39 cm high walls made of a white Plexiglas box. Two black lines were drawn on the floor. Mice were placed into the centre of the arena and allowed to explore the apparatus for 5 min. The number of line crossings (with all four paws placed into a new square) and rearing events (with both front paws raised from the floor) were considered an index of exploratory behaviour. The instrument was cleaned with 1% ethanol to remove scent clues after each trial.

#### Tail suspension test (TST)

This procedure was based on the method described previously (Lin et al. 2014). Experiments were performed under acoustic and visual isolation. Mice were suspended 20 cm above the floor using adhesive tape placed 3 cm from the tip of the tail and at least 15 cm away from the nearest objects. Animals were allowed to hang for 6 min and the duration of immobility was recorded during the last 4 min. Immobility was defined as the complete cessation of movement while suspended. The immobility time was recorded by an observer blind to the treatment.

### Preparation of plasma and brain tissue samples

After the last behavioural test, the animals were immediately sacrificed for biochemical assays. Blood samples (about 500 μL) were collected in heparinized 1.5 mL-polythene tubes immediately, then centrifuged at 3500 rpm for 10 min at 4 °C. The plasma obtained was stored at −20 °C until analysis.

After the blood was obtained, brains were rapidly removed, quickly rinsed in phosphate buffered saline and flash frozen in liquid nitrogen. The brain tissues were stored at −80 °C until use.

### Sample pretreatment

Plasma samples: Mouse plasma samples aliquots (100 μL) were spiked with 10 μL of IS solution (0.2 mg/mL) and deproteinized by addition of 90 μL 0.4 M perchloric acid, then vortexed for 30 s. The samples were then centrifuged at 13,000 rpm at 4 °C for 10 min. The clean upper layer (100 μL) was diluted with 900 μL 40 mM sodium borate buffer.

Brain tissue samples: Mouse brain weighing 10–20 mg was homogenized in a disposable glass tube after addition of ice cold Normal saline (1 g: 9 mL). Brain homogenate (20 μL) was added to 10 μL IS (0.4 mg/mL, methanol), and deproteinized by addition of 70 μL 0.4 M perchloric acid, then vortexed for 30 s. The samples were then centrifuged at 13,000 rpm at 4 °C for 10 min. The clean upper layer (50 μL) was diluted with 950 μL 40 mM sodium borate.

The dilutions were transferred to 2 mL clear snap vial borosilicate after filtrated through a 0.22 μm filter. A 5 μL aliquot was then injected into the HPLC system for analysis.

### Analytical procedures

#### Liquid chromatographic and fluorescence spectrometric conditions

Mobile phase A consisted of 10 mM disodium hydrogen phosphate buffer and 10 mM sodium borate buffer in water (pH 10.6). Mobile phase B was methanol and Mobile phase C was acetonitrile. The mobile phase consisted of 78% A, 13% B and 9% C. The flow rate was 1.0 mL/min and the injection volume was 5 μL. Fluorescence detector was used operated at 355 nm (excitation) and 450 nm (emission), respectively. The autosampler was kept at 4 °C and column temperature was 35 °C.

#### Preparation of working solutions and calibration curves

Stock solutions of Glu, Gln and GABA at 2.0 mg/mL were prepared by dissolving 2.12 mg Glu, 2.00 mg Gln and 2.37 mg GABA in 10 mL of 50% methanol. IS was prepared in methanol at a target concentration of 2.0 mg/mL. The stock solutions were then diluted with 50% methanol to generate a series of standard solutions. Finally, the calibration standards of three amino acids were prepared at concentrations shown in Table 1, the final concentration of IS solution was 800 ng/mL.

**Table 1.** The concentration of amino acids in standard curve.

Standard substances (μg/mL)	1	2	3	4	5	6	7
Glu	0.10	0.20	0.40	0.80	1.6	3.2	6.4
Gln	0.10	0.20	0.40	0.80	1.6	2.4	3.2
GABA	0.05	0.10	0.20	0.40	0.80	1.0	1.6

#### Assay validation

As the analyte was endogenous, the linearity and sensitivity of the assay were validated by using respective standards spiked of IS. The calibration curves for each analyte were obtained by linear regression analysis with 1/x^2^ weighting factor, and plotting the peak area ratio (y) of analytes to the IS *versus* the nominal concentration (*x*) of analytes. The limit of detection (LOD) was determined as signal-to-noise ratio >3 and the limit of quantification (LOQ) was measured as signal-to-noise ratio >10.

The blank samples were prepared according to the approach described above in ‘*Sample pre-treatment*’ QC samples were prepared by addition of three different levels of standards into the blank samples (*n* = 6/level).

The intra-day precision and accuracy were assessed by analysis of neurotransmitters in QC samples (*n* = 6) on the same day. Inter-day precision was evaluated by repeated analysis of neurotransmitter in QC samples over three consecutive days. The minimum acceptance criterion for the intra- and inter-day RSD of the calibration standards is within ±15% of the nominal concentration.

The recovery rate of each analyte was calculated by comparing the difference between QC and blank samples with the added standard. And the accuracy was within 80–120%, all compared to the correspondent nominal concentration.

The stability of three neurotransmitters was evaluated under different storage conditions. In this study, the stability of QC samples after being stored at 4 °C for 24 h and experienced three freeze-thaw cycles were investigated.

### Statistics analysis

Data are presented as mean ± standard error of the mean (SEM). Statistical analysis of the data was performed using independent samples *t*-test on GraphPad Prism software version 5.0 (Prism software for PC, GraphPad Software, La Jolla, CA). Values of *p* < 0.05 were considered statistically significant.

## Results and discussion

### Method validation

#### Method development and separation optimization

**Figure 2.**
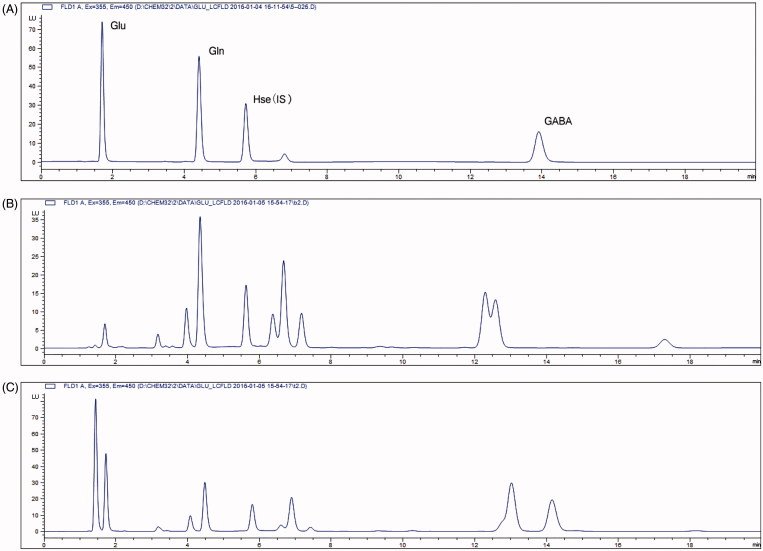
The HPLC chromatogram of standard solution (A). The HPLC chromatogram of mouse blood (B) and brain (C) samples.

To optimize chromatographic conditions, several different columns and various compositions of mobile phase were tested. The derivatives of target amino acids were more stable in pH >9.0 (Penteado et al. 2009). The mobile phase used in this experiment was in pH 10.6, so that HPH-C18 column was selected. Different mobile phase compositions were investigated to provide a good separation condition. Finally, the retention time for targeted analytes was 1.7 min for Glu, 4.4 min for Gln, 13.9 min for GABA and 5.7 min for IS. As displayed in Figure 2, the analytical condition could be used to detect those neurotransmitters in samples successfully.

#### Linearity

After obtaining the values of area under the peak of the six concentrations in triplicate on two separate occasions, a plot of peak area as a function of analyte concentration was developed and the linear regression was calculated by the least squares method. All standards showed good linearity over the concentration range of 100 ng/mL–6.4 μg/mL for Glu, 100 ng/mL–3.2 μg/mL for Gln, and 50 ng/mL–1.6 μg/mL GABA. The correlation coefficient (r) of all analytes was above 0.99. As seen in Table 2, our preliminary data showed that the concentrations of GABA and Glu were higher in mouse brains than in blood.

**Table 2.** Linearity data and quantitation ranges of three neurotransmitters.

Neurotransmitters	Linear range (μg/mL)	Slope^a^	Intercept^a^	Correlation coefficient	LOD (μg/mL)	LOQ (μg/mL)
Glu	0.1–6.4	0.684	0.220	0.999	0.005	0.01
Gln	0.1–3.2	0.711	0.000494	0.999	0.005	0.01
GABA	0.05–1.6	1.078	−0.0241	0.996	0.001	0.005

a Parameters for linear regression equation of *y* = *kx* + *c*, where the value of slope equals to *k* and that of the intercept equal to *c*.

#### Accuracy, stability, recovery and precision

The accuracy of the assay was estimated for each QC sample by comparing the measured concentration to the actual concentration. The results of the accuracy were summarized in Table 3.

**Table 3.** Results of precision and accuracy analysis.

Neurotransmitters	Added (μg/mL)	Intra precision (RSD%)	Inter precision (RSD%)	Accuracy (mean ± SD%)
Glu	0.20	10.3	2.65	103.5
	2.0	6.9	1.45	105.1
	4.8	5.2	2.88	103.9
Gln	0.20	4.5	4.44	107.5
	1.0	8.0	1.32	102.3
	2.4	5.5	4.14	101.3
GABA	0.10	13.2	10.76	99.6
	0.80	7.5	0.98	97.9
	1.2	7.7	3.15	102.9

Data are expressed as mean ± SEM, *n* = 6.

The stability of the sample solutions was checked by reanalysing these solutions on subsequent days. Twenty-four hours after preparation, the signal corresponding to all neurotransmitters was not decreased. And there was no significant degradation observed under freeze-thaw cycles.

The recovery experiment with the spiked samples, conducted in six replicates. The results of the recovery and precision study were summarized in Table 4. The results showed that the recovery rate of all the analytes at three concentrations examined varied from 86.1 to 112.5% that fell within the acceptable limits. The data of intra-day (*n* = 6) and inter-day (*n* = 3) precision and accuracy of the method evaluated from QC samples were in a reasonable range as the RSD of which did not exceed 15%. The results indicated that the present method had a satisfactory accuracy and precision. Taken together, the method which we had developed was proven to be reliable for routine analysis.

**Table 4.** Extraction recovery of Glu, Gln and GABA.

Added (μg/mL)		Plasma		Brain	
		Recovery (%)	RSD (%)	Recovery (%)	RSD (%)
Glu	2.0	112.47 ± 6.59	5.86	95.48 ± 3.85	4.03
Gln	1.0	86.14 ± 13.04	15.14	86.31 ± 1.48	1.72
GABA	0.80	103.42 ± 7.37	7.13	99.64 ± 1.37	1.37

Data are expressed as Mean ± SEM, *n* = 6.

### Determination of neurotransmitters in CUMS mouse plasma and brain

**Figure 3.**
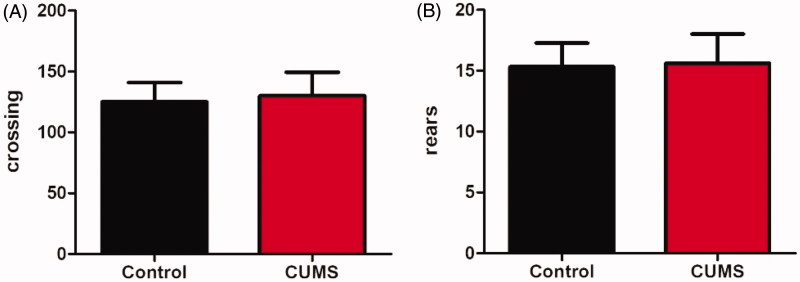
Effect of CUMS on the locomotor activity. The crossing (A) and rears (B) of CUMS model mice (B). Data are expressed as Mean ± SEM, *n* = 8–10.

**Figure 4.**
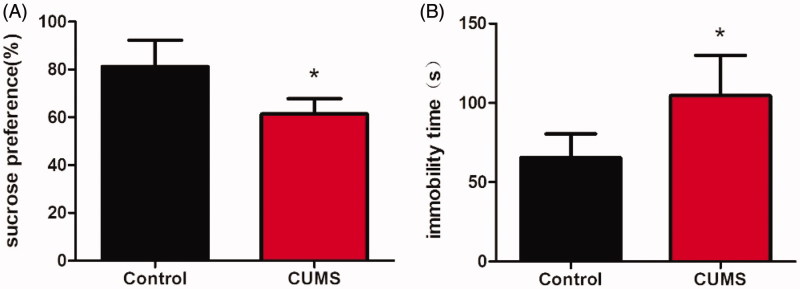
The depression-like behavior of mice induced by CUMS. Changes of the sucrose preference in CUMS mice (A) and the immobility time in tail suspended test of mice after CUMS (B). Data are expressed as Mean ± SEM, *n* = 8–10. *p < 0.05.

After CUMS stimulated for 8 weeks, the depression-like behaviour of mice was assessed using OFT, SPT and TST successively. We examined the effects of CUMS on locomotor activity by submitting the animals to OFT. As shown in Figure 3, the mice locomotor activity measured by line crossing and rearing did not alter. Among all behavioural tasks we used in the current work, the SPT was most commonly used to examine depressant-like behaviours anhedonia (Serchov et al. 2016), the inability to experience pleasure from rewarding or enjoyable activities is the core symptom of depression (Moreau 1997). After the establishment of CUMS model, a reduction in the sucrose preference ratio in experimental relative to control mice was observed (Figure 4(A), *p* <0.05). TST was widely used as an animal model of human depressive disorders; characteristic behaviour scored in the test was termed immobility, reflecting a behavioural state of despair, as seen in human depression (Steru et al. 1985). In this test, immobile time of CUMS mice was longer than that of the control mice (Figure 4(B), *p* <0.05). The results of SPT and TST both showed CUMS mice displayed significant depression-like behaviours.

**Figure 5.**
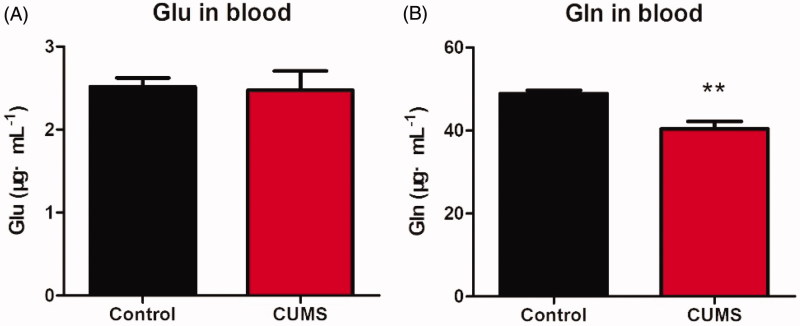
The concentration of Glu and Gln in mouse plasma. The Glu contained in plasma (A); The Gln contained in plasma (B). Data are expressed as Mean ± SEM, *n* = 8–10. **p < 0.01.

**Figure 6.**
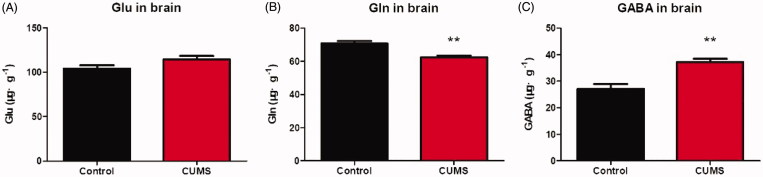
The concentration of Glu, Gln and GABA in mouse brain. The Glu contained in brain (A); The Gln contained in brain (B); The GABA contained in brain (C). Data are expressed as Mean ± SEM, *n* = 8–10. **p < 0.01.

After behavioural tests, all mice were sacrificed; the plasma and brain were collected and subjected to further neurotransmitter analysis. As shown in Figures, the contents of Glu measured in depression mice did not differ from that in control mice in both plasma (Figure 5(A)) and brain tissues (Figure 6(A)). The Gln was significantly decreased in both plasma (Figure 5(B), *p* < 0.01) and brain tissues (Figure 6(B), *p* <0.01). And the GABA in brain was significantly elevated (Figure 6(C), *p* < 0.01), while the GABA in plasma was not detected. As we known, GABA is the chief inhibitory neurotransmitter in the mammalian central nervous system, it may responsible for depression (Bradley et al. 2018). Importantly, Gln in plasma and brain were simultaneously detected in our method, Gln is the most abundant amino acid in your body and plays a number of important biological functions. It has been found that l-Gln supplementation showed antidepressant properties on adult study participants suffering from depression (Cocchi 1976).

The blood-brain barrier (BBB) is a highly selective semipermeable membrane barrier that separates the circulating blood from the brain (Daneman and Prat 2015), it provides an optimal chemical environment for cerebral function. Studies showed that the BBB is impermeable to Glu, even at high concentrations (Hawkins 2009; Hawkins and Viña 2016). As we had detected, the concentrations of Glu in brain is much higher than plasma, so that the blood contents cannot stand for the level of Glu in the brain of patients with depression. The GABA acts as an inhibitory neurotransmitter, researches had shown that GABA was closely associated with depression (Hashimoto et al. 2016; Küçükibrahimoğlu et al. 2009; Mohler 2012). We also detected an increase GABA in the brain of CUMS mice, but GABA in plasma was not detected. It may own to the low sensitivity of fluorescence detector, but this method is more simple and low-cost. And we found that the GABA cannot cross through the BBB (Abbott et al. 2010), so it is not necessary to determine the content of GABA in plasma. Glu is the most abundant amino acid in plasma and cerebrospinal fluid and a precursor for the main central nervous system excitatory (Glu) and inhibitory (GABA) neurotransmitters, and it is one of the few amino acids that can directly cross the BBB (Brosnan 2003). In our experiment, the Gln decreased in both in plasma and brain of depressant mice, which provides a more objective indicator in blood for evaluation of depression extent.

## Conclusions

A convenient, fast and reliable HPLC method, considered as an accurate assay for the simultaneous determination of the levels of amino acids in the Glu-Gln cycling-Glu, Gln and GABA in mouse brain and plasma, has been developed. We believe that this method will be especially useful in the research of neurological diseases with the altered amino acids in brain and plasma.
